# Inflorescence Development and the Role of *LsFT* in Regulating Bolting in Lettuce (*Lactuca sativa* L.)

**DOI:** 10.3389/fpls.2017.02248

**Published:** 2018-01-18

**Authors:** Zijing Chen, Yingyan Han, Kang Ning, Yunyu Ding, Wensheng Zhao, Shuangshuang Yan, Chen Luo, Xiaotang Jiang, Danfeng Ge, Renyi Liu, Qian Wang, Xiaolan Zhang

**Affiliations:** ^1^Department of Vegetable Science, Beijing Key Laboratory of Growth and Developmental Regulation for Protected Vegetable Crops, China Agricultural University, Beijing, China; ^2^New Technological Laboratory in Agriculture Application in Beijing, College of Plant Science and Technology, Beijing University of Agriculture, Beijing, China; ^3^Shanghai Center for Plant Stress Biology, Shanghai Institutes for Biological Sciences, Chinese Academy of Sciences, Shanghai, China

**Keywords:** lettuce, morphology, floral transition, *LsFT*, bolting

## Abstract

Lettuce (*Lactuca sativa* L.) is one of the most important leafy vegetable that is consumed during its vegetative growth. The transition from vegetative to reproductive growth is induced by high temperature, which has significant economic effect on lettuce production. However, the progression of floral transition and the molecular regulation of bolting are largely unknown. Here we morphologically characterized the inflorescence development and functionally analyzed the *FLOWERING LOCUS T (LsFT)* gene during bolting regulation in lettuce. We described the eight developmental stages during floral transition process. The expression of *LsFT* was negatively correlated with bolting in different lettuce varieties, and was promoted by heat treatment. Overexpression of *LsFT* could recover the late-flowering phenotype of *ft-2* mutant. Knockdown of *LsFT* by RNA interference dramatically delayed bolting in lettuce, and failed to respond to high temperature. Therefore, this study dissects the process of inflorescence development and characterizes the role of *LsFT* in bolting regulation in lettuce.

## Introduction

Lettuce (*Lactuca sativa* L.) is one of the most important leafy vegetables that cultivated worldwide and consumed throughout the year (Fukuda et al., 2009). In 2013, the world production of lettuce and chicory was 24896 tons^1^. Lettuce belongs to the Asteraceae family, and is a self-fertilizing diploid plant with 2n = 2x = 18 chromosomes and an estimated 2.5 Gb genome size (Reyes-Chin-Wo et al., 2017). Based on plant morphology, leafy lettuce can be classified into four types: romaine, iceberg, butterhead and non-heading (Simko et al., 2013). In addition to its easy preparation for consumption, lettuce is enriched in health-promoting compounds such as vitamins C and E, polyphenols, fibers, tocopherols and lutein (Hooper and Cassidy, 2006), which play important roles in preventing the incidence of many chronic diseases (Christopoulou, 2016). Another striking feature of lettuce is that bolting (rapid stem elongation) is promoted by high temperature (Fukuda et al., 2011). Upon bolting, leafy lettuce becomes bitter and unsaleable. Thus bolting resistance is an essential desirable trait in lettuce breeding, especially for cultivation during the hot summer or in tropical regions. However, the progression of inflorescence development and the molecular regulation of bolting remain largely elusive in lettuce.

The transition to flowering is the process by which flowering plants switch from vegetative to reproductive growth. This transition involves a specialized structure called the shoot apical meristem (SAM), which is situated at the tip of the shoot apex and is comprised of a pool of stem cells that continuously divide and replenish themselves (Fletcher, 2002). During the vegetative stage, the SAM produces leaves in a predictable pattern from the peripheral zone. After transition, the SAM elongates and changes into the inflorescence meristem (IM), which produces flowers during the reproductive phase (Kobayashi et al., 2012). Unlike most species producing single flowers, many members in the Asteraceae family have composite flowers in the form of capitula surrounded by involucral bracts. Each capitulum is generally made up of tens to hundreds of florets with specialized structure and function (Harris, 1995).

In *Arabidopsis*, floral transition is regulated by six genetic pathways that incorporate important endogenous and environmental cues, including vernalization, photoperiod, gibberellin (GA), autonomous, ambient temperature and age pathways (Fornara et al., 2010; Srikanth and Schmid, 2011). The molecular interpretation of these flowering signals converge at three transcription factors, *FLOWERING LOCUS T* (*FT*), *SUPPRESSOR OF OVEREXPRESSION OF CONSTANS1* (*SOC1*) and *LEAFY* (*LFY*) (Balasubramanian et al., 2006). *FT* belongs to the phosphatidylethanolamine-binding protein (PEBP) family, which consists of six members in *Arabidopsis*-48pc: *FT, TERMINAL FLOWER 1* (*TFL1*), *ARABIDOPSIS THALIANA CENTRORADIALIS* (*ATC*), *BROTHER OF FT AND TFL1* (*BFT*), *MOTHER OF FT AND TFL1* (*MFT*) and *TWIN SISTER OF FT* (*TSF*) (Kardailsky et al., 1999; Yamaguchi et al., 2005). *FT* is produced in the leaves under favorable flowering conditions, and moves from the phloem to the shoot apex where it binds to the bZIP transcription factor *FLOWERING LOCUS D* (*FD*) to activate downstream genes such as *APETALA1* (*AP1*) and *LEAFY* (*LFY*) (Corbesier et al., 2007; Mathieu et al., 2007). In *Arabidopsis*, FT and TFL1 have opposite roles in determining flowering time, and their antagonistic action depends on the presence of special amino acid residues, with Tyr85/Gln140 in FT and His88/Asp144 in TFL1 (Hanzawa et al., 2005; Ahn et al., 2006). Homologs of *FT* genes have been characterized in many plant species. For example, three *FT-like* genes *CsFTL1, CsFTL2* and *CsFTL3* were isolated from *Chrysanthemum seticuspe*. Overexpression of *CsFTL3* in *Chrysanthemum seticuspe* induce flowering under non-inductive conditions (Oda et al., 2012). In sunflower, a frame shift mutation in *HaFT1* delayed flowering through interference with the action of another *FT* paralog *HaFT4* (Blackman et al., 2010). The putative lettuce *FT* homolog *LsFT* was isolated as well, and heterologous expression of *LsFT* promoted flowering in wild-type *Arabidopsis* (Fukuda et al., 2011). However, functional characterization of *LsFT* in regulation of lettuce flowering is still lacking.

Herein, we investigated the histological and morphological features of capitulum development in lettuce, and explored the expression of *LsFT* in nine lettuce varieties with different bolting times. Further, knockdown of *LsFT* by RNA interference resulted in significant delay in lettuce bolting and *LsFT* knockdown lines failed to respond to high temperature, indicating the important role of *LsFT* in regulating bolting in lettuce.

## Materials and Methods

### Plant Materials and Growth Conditions

The leafy lettuce (*Lactuca sativa* L.) varieties S24, S43, S7, S1, S3, S8, S28, S26, and S39 were selected from 705 lettuce collection for their different bolting times and were grown in the Beijing University of Agriculture Experimental Station under standard greenhouse conditions. Pest control and water management were performed according to standard practices. For morphological characterization and transgenic analysis, lettuce variety S39 (bolting sensitive) was cultivated in a growth chambers at 25/15°C (day/night), and using a 16 h day/8 h night with a photon flux density (PFD) of 200 μmol photons m^-2^s^-1^. The wild type and *LsFT-*RNAi lettuce were planted in a growth chambers at 25/15°C (day/night) under a 16 h day/8 h night cycle. The *Arabidopsis ft-2* mutant was obtained from The *Arabidopsis* Information Resource^2^. All *Arabidopsis* plants were grown in soil at 22°C under a 16 h/8 h light/dark cycle in the growth chambers.

### Paraffin Sections

Lettuce S39 shoots at different stages of inflorescence development were fixed, embedded, sectioned and dewaxed as previously described (Jiang et al., 2014). Sections (8 μm thick) were mounted in neutral resins, and images were taken under a light microscope (D72, Olympus, Tokyo, Japan).

### Scanning Electron Microscopy (SEM)

The shoot apex, inflorescence and florets of different developmental stages were dissected from lettuce S39 under a light microscope (Leica DFC450, Wetzlar, Germany). After dissection, samples were fixed in formaldehyde-acetic acid-ethanol (FAA) overnight, and then critical-point dried in liquid CO_2_, sputter-coated with gold and palladium for 60 s, and visualized at an acceleration voltage of 2 kV using a scanning electron microscope (Hitachi Model S-4700, Tokyo, Japan).

### Gene Cloning

Total RNA was extracted from mature leaves or flower buds using a Quick-RNA isolation Kit (Waryoung, Beijing, China). A TIANGEN reverse transcriptase kit (Tiangen Biotech, Beijing, China) was used to synthesize cDNA. Sequence information of *LsFT* (Lsat_1_v5_gn2_17881.1), *LsAP1* (Lsat_1_v5_gn3_97021.1), *LsAP3* (Lsat_1_v5_gn3_75340.1) and *LsLFY* (Lsat_1_v5_gn4_ 84380) were obtained by homologous alignment in the lettuce website^3^. *AP1, AP3*, and *LFY* were the downstream genes of *FT* (Jack et al., 1992; Wagner et al., 1999; Ferrandiz et al., 2000). Coding sequences (CDS) of *LsAP1, LsAP3* and *LsLFY* were cloned from the shoot apex of bolting-sensitive S39 line and LsFT was choned from the mature leaves of S39 using gene specific primers (Supplementary Table S1).

### Quantitative Real-Time PCR

Total RNA was extracted from different tissues of lettuce or *Arabidopsis* using Quick RNA isolation Kit (Waryoung, China). The RNA of wild type and *LsFT-*RNAi lines were extracted from the fourth leaves at 25 days. A TIANGEN reverse transcriptase kit (Tiangen) was used to synthesize cDNA. An ABI PRISM 7500 Real-Time PCR System (Applied Biosystems, Carlsbad, CA, United States) was used for quantitative real-time PCR (qRT-PCR) experiments. Three biological replicates and three technical replicates (3 × 3) were performed for each gene. Lettuce 18srRNA (Gene Bank accession number HM047292.1) and *Arabidopsis* ACTIN2 (Gene Bank accession number AT3G18780.2) genes were used as internal controls to normalize expression data. The gene-specific primers are listed in Supplementary Table S1.

### Subcellular Localization

The open reading frame (ORF) of *LsFT* cDNA was amplified and introduced into the XbaI and SmaI sites fused with GFP in a pUC-19 vector. The 35S promoter was used for directing the expression of fusion gene. Subcellular localization of LsFT by fusion with green fluorescent protein (GFP) in the C-terminal region The bombardment of onion epidermal cells was performed as previously described (Dong et al., 2013), and images were taken using a confocal laser-scanning microscope (Carl Zeiss LSM 510, Germany) excited at a 488 nm wavelength.

### Heat Treatment

Lettuce varieties S24, S43, S7, S1, S3, S8, S28, S26, S39 were used for heat treatment. The lettuce plants were grown in the growth chamber at 25°C /15°C (16 h day/8 h night) for 28 days, and then moved to the growth chamber at 35°C/25°C (16 h day/8 h night) for heat treatment. After 48 h, the fourth leaves were cut off and frozen in liquid nitrogen, and stored at -80°C until further use. The WT and *LsFT*-RNAi lines were grown for 28 days in the growth chamber at 25°C/15°C (16 h day/8 h night), and then moved to the growth chamber at 35°C /25°C (16 h day/8 h night) for heat treatment. Upon 1, 2, 3, and 4 Day after heat treatment, the fourth leaves were harvested at 10 am in the morning and frozen in liquid nitrogen, and stored at -80°C until further use.

### Phylogenetic Analysis

The amino acid sequences of LsFT (Lsat_1_v5_gn_2_17881.1), HaFT2 (GQ884982), HaFT4 (GQ884984), CsFTL1 (AB679270), CsFTL2 (AB679271), CsFTL3 (AB679272), OsRFT1 (AB426873.1), OsHd3a (AB052944), AtFT (AT1G65480.1), AtTSF (AT4G20370.1), CiFT (AB027456), BvFT1 (HM448910.1), BvFT2 (HM448912.1), PnFT2a (AB109804.1), PnFT4a (AB369074.1), GhFT1 (HM631972), MdFT (AB161112) were obtained from the National center for Biotechnology Information website. The phylogenetic tree was constructed using the Neighbor-Joining (NJ) method with default parameters in the MEGA 6.0 software. The numbers next to nodes are 1,000 bootstraps (Tamura et al., 2013).

### Ectopic Expression of *LsFT* in *Arabidopsis*

To generate the *LsFT* overexpression construct, full length *LsFT* CDS were amplified and cloned into the binary vector pBI121 through XbaI and SmaI sites. The construct was then introduced into Agrobacterium strain C58 by electroporation and transformed into *Arabidopsis ft-2* mutant plants using the floral-dip method (Clough and Bent, 1998). Transgenic plants were screened on Murashige and Skoog (MS) medium with 40 mg/L kanamycin. Primer information are listed in Supplementary Table S1.

### Agrobacterium-Mediated Transformation in Lettuce

To obtain *LsFT*-RNAi transgenic plants, the 178-bp sense and antisense fragments from the 3′ end of *LsFT* were amplified using gene specific primers containing AscI(5′ end)/SwaI(3′ end) and BamHI(5′ end)/SpeI(3′ end) sites, respectively. Primers containing restriction enzyme cutting sites are listed in Supplementary Table S1. The two fragments were inserted into a pFGC1008 vector, and an empty pFGC1008 vector was used as a transformation control. Both the resultant *LsFT*-RNAi construct and empty pFGC1008 vector were then delivered into Agrobacterium by electroporation.

Cotyledon transformation of S39 lettuce variety was performed as described previously with modifications (Lee et al., 2007). The brief procedure is described as follows: mature lettuce seeds were sterilized with 30% sodium hypochlorite for 5 min, and sown on MS medium (Supplementary Figure S3A). When the seedlings were 6 days old (Supplementary Figure S3B), cotyledons were cut by surgical blade, and incubated in ½ MS liquid medium containing *Agrobacterium tumefaciens* cells with an optical density (OD) of 0.2–0.3 for 13 min. Next, the cotyledons were placed on a piece of filter paper on top of co-cultivated medium and co-cultivated at 28°C (dark) for 2 days (Supplementary Figure S3C). Subsequently, cotyledons were transferred to differentiation medium; pale green calli were produced within 1–2 weeks with green shoots forming from them (Supplementary Figure S3D). When the green shoots were at the three-leaf stage, they were transferred to shoot-inducing medium (Supplementary Figure S3E). After 2 weeks, the regenerated shoot was transferred to root-inducing medium (Supplementary Figure S3F), with roots forming 2 weeks after transfer (Supplementary Figure S3G). The resultant regenerated plant was acclimated (removing the lid to allow access to air) for 1 week, and transferred to soil under standard growth conditions (Supplementary Figure S3H). The transformation efficiency was around 10% (number of positive transformants/number of regenerated plants) in S39. Recipes for regeneration mediums are as follows: co-cultivating medium [MS medium supplemented with 0.1 mg/L 1-naphthlcetic acid (NAA) and 0.1 mg/L 6-Benzylaminopurine (6-BA), pH 6.5–6.8], differentiation medium (MS medium supplemented with 75mg/L chloramphenicol, 300 mg/L carbenicillin, 0.1mg/L NAA, 0.1mg/L 6-BA, pH 6.5–6.8), shoot-inducing medium (1/2 gellan MS medium supplemented with 300 mg/L carbenicillin), and root-inducing medium (1/2 MS medium supplemented with 300 mg/L carbenicillin).

### RNA Extraction, Amplification and RNA-Seq Library Construction

Total RNA from LCM samples of S1, S2, S3, S4 Stage were extracted using the Arcturus PicoPure RNA isolation Kit (Applied Biosystems) in conjunction with DNase I (Qiagen) for removing potential DNA contamination. The Target Amp 2-Round Aminoallyl-aRNA Amplification Kit 1.0 (Epicentre Biotechnologies) was used for RNA amplification following the manufacturer’s protocol. RNA abstracted from LCM was subjected to two rounds of amplification to yield 35–55 μg of RNA per sample. Next, RNA-Seq were made using NEB Next Ultra Directional RNA Library Prep Kit for Illumina (NEB, Ispawich, MA, United States). The libraries were pooled and sequenced in one lane with 100 bp paired-end reads on the Illumina HiSeq 2000. Sequencing data were deposited to the Sequence Read Archive (SRA) at the National Center for Biotechnology Information (NCBI) with accession number GSE108260.

## Results

### Shoot Development and Capitulum Structure in Lettuce

**FIGURE 1:**
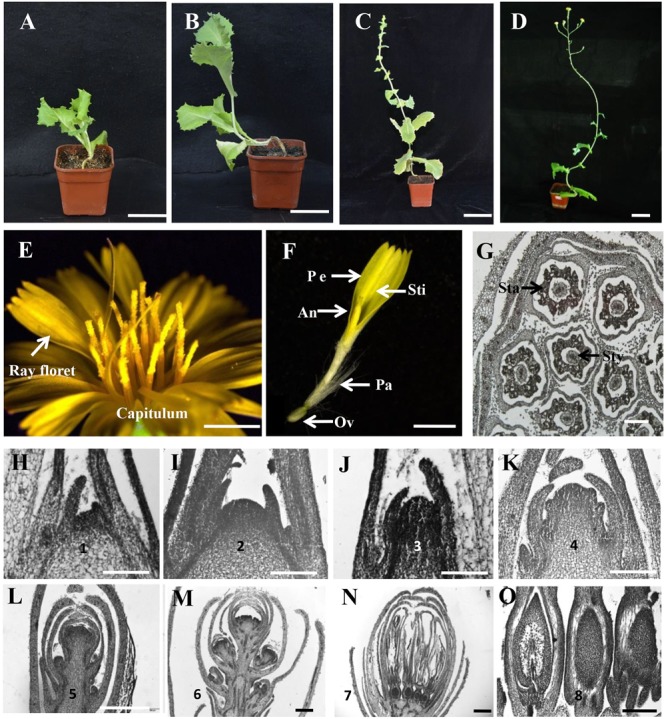
Capitulum structure and floral transition in lettuce. **(A–D)** The four developmental stages of lettuce. From left to right, vegetative stage, bolting stage, inflorescence stage, and flowering stage. **(E)** The morphology of the capitulum in lettuce. **(F)** The structure of a ray floret. An, anther, Ov, ovary; Pe, petal; Sti, stigma; Pa, pappus. **(G)** The transverse section of a capitulum in lettuce. Sta, stamen; Sty, style. **(H–O)** Histological sections showing the stages of floral transition progression in lettuce. **(H)** Stage 1 vegetative shoot apical meristem (SAM) (20 DAP [days after planting]). **(I)** Stage 2 dome-shaped SAM (28 DAP). **(J)** Stage 3 elongated meristem showing the transition from vegetative to reproductive growth (35 DAP). **(K)** Stages 4 indicating the development of involucre primordia from the inflorescence meristem (IM) (45 DAP). **(L)** Stage 5 showing the development of capitulum primordia from the IM (55 DAP). **(M)** Stage 6 highlighting the development of floret primordia (75 DAP). **(N)** Stage 7 indicating the formation of ray floret (85 DAP). **(O)** Stage 8 showing the ovary development (95 DAP). Scale bars represent 6 cm in **(A–D)**, 2 mm in **(E,F)**, 200 μm in **(G)**, and 50 μm in **(H–O)**.

To dissect the process of flowering in lettuce, a bolting-sensitive variety S39 was used and grown in a growth chamber at 25/15°C (day/night). The whole life cycle of S39 takes about 130 days, in which four critical developmental stages can be observed: vegetative stage (0–35 DAP [days after planting]), bolting stage (35–75 DAP), inflorescence stage (75–95 DAP), and flowering stage (95–125 DAP) (**Figures 1A–D**). Generally, the bolting stage starts from the 7–8 leaf stage, and the inflorescence stage starts upon the shoot producing 10–12 elongated internodes. The lettuce capitula are complex inflorescences. Each capitulum is made up of 15–25 ray florets. Each ray floret is composed of a ligule corolla with five fused petals, five fused anthers that form a tube surrounding the style and the bipartite stigma, a modified calyx called a pappus, and an ovary that produces one seed after pollination (**Figures 1E–G**). The blossom of each capitulum lasts for 1–2 h, and seeds are ready for harvest 2 weeks after anthesis.

### Progression of Floral Transition in Lettuce

To characterize the floral transition progression in lettuce, we chose the S39 line growing in the growth chamber at 25/15°C (day/night) for observation. Histological sections and morphological features were recorded by optical microscope and scanning electron microscope (SEM) over time. As shown in **Figure 1**, floral transition in lettuce can be divided into eight stages. Stage 1 (20 DAP [days after planting]) with a flat shoot apical meristem (SAM) (**Figure 1H** and Supplementary Figures S1D,J) and stage 2 (28 DAP) with a dome-shaped SAM (**Figure 1I** and Supplementary Figures S1E,K). We considered stage 1 and 2 are the vegetative stage because in the bolting resistant line S24, both stage 1 and stage 2 are observed much earlier before bolting, with stage 1 extended to 35 DAP, and stage 2 from 45 to 65 DAP (Supplementary Figures S1A–C). In line S39, stage 3 (35 DAP) marks the transition from vegetative to reproductive growth with elongated SAM (**Figure 1J**). Stage 4 (45 DAP) highlights the development of involucre primordium from inflorescence meristem (IM) (**Figure 1K** and Supplementary Figures S1F,G,L). Stage 5 (55 DAP) is featured by the appearance of the capitulum primordia (**Figure 1L** and Supplementary Figures S1H,M). By stage 6 (75 DAP), more and more capitulum primordia were produced and the top capitulum initiates floret primordia (**Figure 1M** and Supplementary Figures S1I,N,O). Stage 7 (85 DAP) represents the formation of ray florets (**Figure 1N**), and stage 8 (95 DAP) marks the ovary development which located in the base of each floret and gives rise to the dry seed measuring 3–4 mm long at maturity (**Figure 1O**).

### Expression Analysis and Subcellular Localization of *LsFT* in Lettuce

**FIGURE 2:**
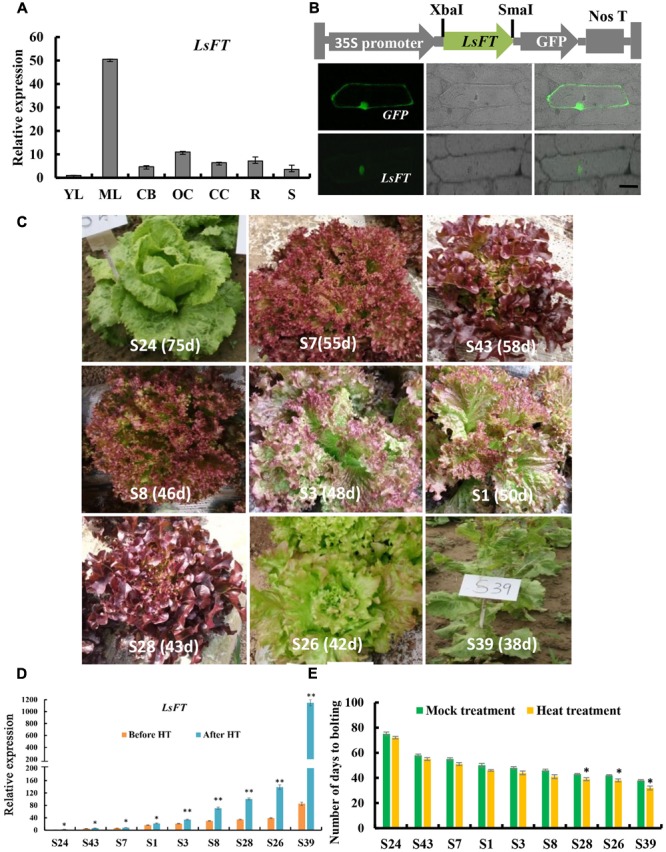
Expression analysis and subcellular localization of *LsFT* in lettuce. **(A)** Quantitative real time RT-PCR (qRT-PCR) analysis of *LsFT* in different tissues of lettuce. YL, young leaves; ML, mature leaves; CB, capitulum buds; OC, opening capitulum; CC, closing capitulum; R, root; S, stem. Lettuce 18S ribosomal RNA (HM047292.1) was used as an internal reference to normalize the expression data. **(B)** The top row is the diagram of the fusion protein construct used for subcellular localization. The open reading frame (ORF) of *LsFT* cDNA was introduced into PUC-19 vector using the XbaI and SmaI sites, and fused with GFP in frame. The 35S promoter directs the expression of fusion genes. The bottom row is the subcellular localization of LsFT fusion protein in onion epidermal cells. Plasmid with green fluorescent protein (GFP) alone served as the control (top). Scale bar represents 50 μm. **(C)** The morphology of lettuce varieties with different bolting times. The days to bolting in the growth chamber were as follows: S24 (75 days), S43 (58 days), S7 (55 days), S1 (50 days), S3 (48 days), S8 (46 days), S28 (43 days), S26 (42 days), S39 (38 days). **(D)** qRT-PCR analysis of *LsFT* in different lettuce varieties before and after heat treatment (35°C/25°C) for 48 h. **(E)** The number of days to bolting under heat treatment (35°C/25°C) and mock treatment. Error bars represent standard errors. Significant difference were determined by student’s *t*-test (^∗^represents *P* < 0.05 and ^∗∗^indicates *P* < 0.01).

Previous studies shown that *FT* gene plays a key role in promoting flowering in several plant species, and that overexpression of the putative lettuce *FT* homolog, *LsFT*, promotes flowering in wild-type *Arabidopsis* (Kotoda et al., 2010; Fukuda et al., 2011; Hecht et al., 2011). Phylogenetic analysis showed that the FTs in the Asteraceae family formed into a subclade (blue lines in Supplementary Figure S2). Unlike Chrysanthemum or Helianthus that has three functional FT genes (Blackman et al., 2010; Oda et al., 2012), sequence analysis indicated that there is only one *LsFT* gene in lettuce that shares the highest similarity with CsFTL3 in Chrysanthemum. To further characterize the *LsFT* function, we examined the expression of *LsFT* in various tissues of lettuce, including young leaves, mature leaves, capitulum buds, opening capitulum, closing capitulum, roots and stems by quantitative real time PCR (qRT-PCR) analysis (**Figure 2A**). *LsFT* was shown to be highly expressed in the mature leaf (**Figure 2A**), which was consistent with previous findings (Fukuda et al., 2011; Xiang et al., 2012). Subcellular localization of LsFT by fusion with GFP in the C-terminal region showed that LsFT is localized to the nucleus (**Figure 2B**). To explore whether the expression of *LsFT* correlates with bolting in lettuce, qRT-PCR of *LsFT* were performed in the mature leaves of nine lettuce varieties, which were selected from 705 lettuce varieties with different bolting times (**Figure 2C**). Specifically, the nine lettuce varieties were divided into three groups: late bolting (S24, S43, S7), middle bolting (S1, S3, S8) and early bolting (S28, S26, S39). The days to bolting (the days to the first visible elongated stem) in the growth chamber were as follows: S24 (75 days), S43 (58 days), S7 (55 days), S1 (50 days), S3 (48 days), S8 (46 days), S28 (43 days), S26 (42 days), S39 (38 days). As shown in **Figure 2D**, the expression of *LsFT* was negatively correlated with the bolting time in lettuce. In the earliest bolting variety S39, *LsFT* transcript accumulation was 85-folds higher than that in the latest bolting variety S24 under normal conditions (**Figure 2D**). Upon heat treatment for 48 h, expression of *LsFT* was promoted in all lettuce varieties, consistent with previous studies (Fukuda et al., 2011). However, the degree of increase was much larger in the early bolting varieties (**Figure 2D**). For example, expression of *LsFT* increased 2.5-folds in the latest bolting variety S24, while 13-folds in the earliest bolting line S39. Consequently, heat treatment significantly promoted bolting in the early bolting varieties, while no dramatic difference were observed in the middle- or late-bolting lines (**Figure 2E**), suggesting the important role of *LsFT* during heat promoted bolting in lettuce.

### Ectopic Expression of *LsFT* Restored the Late-Flowering Phenotype in *ft-2* Mutant *Arabidopsis*

**FIGURE 3:**
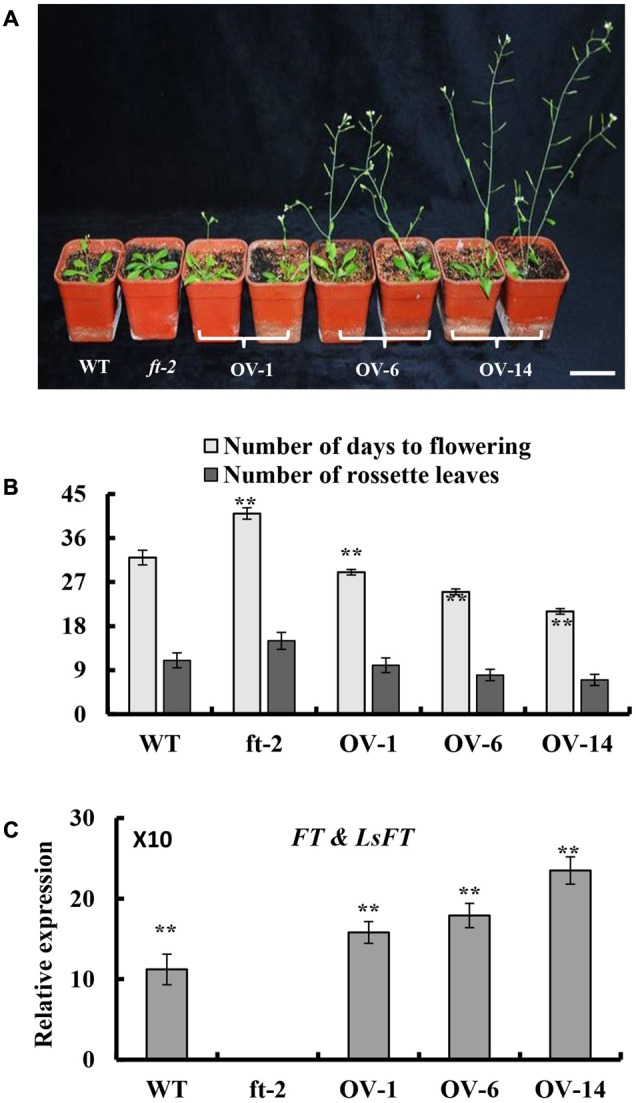
Ectopic expression of *LsFT* in *ft-2* mutant *Arabidopsis* plants. **(A)** Ectopic expression of *LsFT* can fully restore the late-flowering phenotype in *ft-2* mutant plants. **(B)** Quantification of flowering phenotypes in *LsFT* transgenic lines. **(C)** Expression analyses of *FT* and *LsFT* by qRT-PCR in *ft-2*, WT, and overexpression lines of 35S-*LsFT/ft-2* (OV-1, OV-6, OV-14). Error bars represent standard errors. Significant difference were determined by student’s *t*-test (^∗^represents *P* < 0.05 and ^∗∗^indicates *P* < 0.01). Scale bar represents 6 cm.

To explore whether *LsFT* plays an equivalent role in flowering regulation as *Arabidopsis FT*, ectopic expression of *LsFT* under the 35S promoter in the *Arabidopsis ft-2* mutant was performed. A total of 30 transgenic lines were obtained and all lines flowered earlier than the *ft-2* mutant plants (**Figure 3A**). Based on the severity of the phenotype, transgenic lines could be divided into three classes: Class 1 such as OV-14 line is the strongest, which flowerings 9–11 days earlier than wild-type (WT). Class 2 such as OV-6 line is the moderate, which flowerings 5–7 days earlier than WT. Class 3 such as OV-1 line is the weakest, which flowerings at about the same time as WT and 9–11 days earlier than *ft-2* mutant plants (**Figure 3B**). Interestingly, qRT-PCR analyses showed that the severity of the phenotype positively correlated with the expression of *LsFT* in the transgenic lines (**Figure 3C**), suggesting that *LsFT* can fully replace the function of *FT* in promoting flowering in *Arabidopsis*.

### Functional Characterization of *LsFT* in Lettuce

**FIGURE 4:**
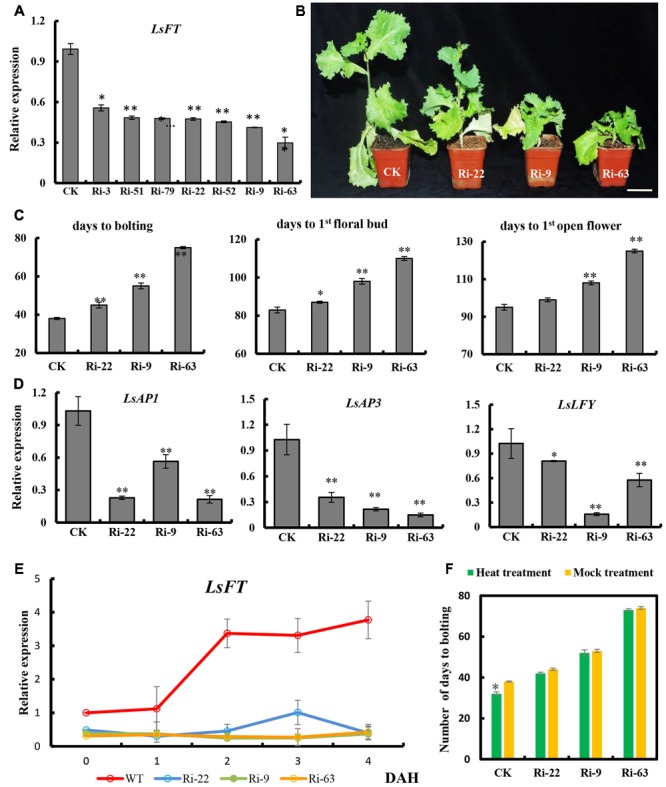
Functional characterization of *LsFT* in lettuce. **(A)** qRT-PCR analysis of *LsFT* expression in different *LsFT-RNAi* lines in lettuce. **(B)** *LsFT-RNAi* resulted in significant delay in bolting in lettuce. Scale bar represents 6 cm. **(C)** Quantification of the delayed bolting phenotypes in *LsFT-RNAi* lines. **(D)** Expression analyses of *LsAP1, LsAP3* and *LsLFY* in *LsFT-RNAi* lines. Lettuce 18S ribosomal RNA (HM047292.1) was used as an internal reference to normalize the expression data. **(E)** qRT-PCR analysis of *LsFT* in WT and *LsFT-*RNAi lines at 1, 2, 3, 4 days after heat treatment (DAH). **(F)** The number of days to bolting in WT and *LsFT-*RNAi lines under heat treatment and mock treatment. Error bars represent standard errors. Significant difference were determined by student’s *t*-test (^∗^represents *P* < 0.05 and ^∗∗^indicates *P* < 0.01).

To examine the function of *LsFT* in lettuce, we optimized the Agrobacterium-mediated transformation in lettuce (Supplementary Figure S3), and knockdown of *LsFT* by double-stranded RNA interference (*LsFT*-RNAi). Seven independent *LsFT*-RNAi lines were obtained and the expression of *LsFT* decreased to 29–56% in the transgenic lines (**Figure 4A**). Three lines with different expression levels (Ri-22, Ri-9 and Ri-63) were chosen for further characterization. All of the three transgenic lines bolted later than the empty vector control (CK), and the degree of delay positively correlated with the level of *LsFT* knockdown (**Figures 4A,B**). For example, the number of days to bolting was 45, 55, and 75 days in lines Ri-22, Ri-9 and Ri-63, respectively, whereas the CK plants took only 38 days to bolting (**Figure 4C**). Similarly, the appearance of the first floral bud and the first opening flower were significantly delayed in the transgenic lines (**Figure 4C**). These data demonstrated that the decreased *LsFT* expression in the transgenic lines resulted in delayed bolting in lettuce. Next, we examined the transcript abundance of the putative homologs of the *FT* downstream genes, *LsAP1, LsAP3*, and *LsLFY* in the transgenic lines (Jack et al., 1992; Wagner et al., 1999; Ferrandiz et al., 2000). As compared to the control plants, all three genes were significantly down-regulated in the transgenic lines, in which *LsAP3* showed the greatest reduction (43%, 27%, and 17% in lines Ri-22, Ri-9, and Ri-63, respectively) (**Figure 4D**). Furthermore, we explored the response of *LsFT* knockdown lines to heat treatment. In the CK plants, the expression of *LsFT* was significantly increased upon heat treatment, concomitant with precocious bolting. However, *LsFT* knockdown lines failed to respond to heat treatment, with respect to both *LsFT* expression and the number of days to bolting (**Figures 4C,E,F**).

## Discussion

In this study, we characterized the structure of lettuce capitulum and identified the eight developmental stages during floral transition in lettuce (**Figure 1**). Further, we found that *LsFT* was highly expressed in mature leaves, and subcellular localization showed that LsFT was localized to the nucleus (**Figure 2**). Expression of *LsFT* negatively correlated with bolting in different lettuce varieties and was promoted by heat treatment (**Figure 2**). Overexpression of *LsFT* could rescue the phenotype of *Arabidopsis ft-2* mutant (**Figure 3**), and knockdown of *LsFT* by RNAi dramatically delayed bolting in lettuce (**Figure 4**). Moreover, *LsFT* knockdown lines failed to respond to heat treatment, suggesting that *LsFT* may play a role during the heat-promoted bolting in lettuce.

### Capitulum Structure and Floral Transition in Lettuce

Lettuce belongs to the Asteraceae family with unique capitulum structure. Chrysanthemum, Heliantheae and Gerbera hybrid belong to the Asteroideae subfamily; two types of florets (ray and disk) are found in Chrysanthemum and Heliantheae, while three types of florets (ray, trans, disk) are identified in Gerbera hybrid (Broholm et al., 2010; Wang et al., 2014; Liu et al., 2016). Here, we found that the lettuce capitulum is comprised of one type of floret (ray floret).

The floral transition is the process by which flowering plants switching from vegetative growth to productive growth (Alvarez-Buylla et al., 2010). In agricultural crops such as rice, maize and tomato, the products are developed from reproductive organs including seeds and fruits, floral transition is required for crop production, and early flowering is benificial and favored by farmers (Brandstadter et al., 1994; Dong et al., 2012; Kobayashi et al., 2012). However, lettuce is a leafy vegetable whose products come from the vegetative organ (leaf), and floral transition is detrimental for lettuce production. Despite in many species, the floral transition is marked by the SAM shape turning from flat to domed (Brandstadter et al., 1994; Alvarez-Buylla et al., 2010), here we showed that the sign of the transition from vegetative stage to reproductive stage is the elongation of the SAM (stage 3) (**Figure 1**), similar to that in monocot species such as rice and maize (Danilevskaya et al., 2008; Kobayashi et al., 2012). There are two evidences for this notion: (1) both stage 1 and 2 are observed in the bolting resistant line S24, and present much earlier before bolting (Supplementary Figure S1); (2) SAM-specific transcriptome analysis by laser capture microdissection and RNA-Seq showed that there are only 21 differentially expressed genes (DEGs) between stage 2 and stage 1, while 365 DEGs between stage 3 and stage 2, including the two floral marker genes *LsSOC1* and *LsLFY* (Supplementary Table S2) (GSE108260). Thus, stage 2 may be a vegetative state where SAM is competent to integrate inductive signals. The heat insensitivity observed in the late flowering lines may because they were still in stage 1 upon heat treatment and unable to perceive heat or integrate signals for flowering.

### Conservation and Divergence of *FT* Function across Species

Previous studies in many species such as *Arabidopsis*, tobacco, rice and cotton indicated that FT-like proteins were localized in both the cytoplasm and the nucleus (Taoka et al., 2011; Harig et al., 2012; Guo et al., 2015). However, here we found that the *LsFT*-GFP was localized only in nucleus (**Figure 2B**), which is similar to that in grapevine (Yang et al., 2011), implying that the function of FTs may be different in different species.

Previous studies have shown that *FT* is a floral integrator that promotes flowering in many species (Hsu et al., 2006; Klocko et al., 2016). In *Arabidopsis*, the expression of *FT* is induced by the photoperiod pathway through *CONSTANS* (*CO*) (Tiwari et al., 2010). *FLOWERING LOCUS C (FLC*) acts antagonistically to the photoperiod pathway by repressing the key floral integrators, *FT* and *SOC1* (Seo et al., 2009). Within the Asteraceae family, in chrysanthemum, heat treatment resulted in delayed flowering and a decreased expression of *CsFTL3* in leaves (Nakano et al., 2013). Overexpressing of *CsFTL3* in WT *Arabidopsis* flowered earlier than the wild-type plants under SD conditions (Oda et al., 2012). Lettuce *LsFT* was previously shown to promote flowering, but was thought to be functionally unequivalent to *AtFT* because the 35S:*LsFT* lines did not promote flowering as early as 35S:*AtFT* lines in WT *Arabidopsis* (Fukuda et al., 2011). Our results indicated that overexpression of *LsFT* can fully restore the late-flowering phenotype in *ft-2* mutant *Arabidopsis* and played equivalent role as FT in flowering regulation (**Figure 3**). Such discrepancy may lie in the expression level of *LsFT* in different transgenic lines.

Further, we found that the expression of *LsFT* negatively correlated with bolting time in different lettuce varieties, and the expression of *LsFT* was increased much higher in early bolting lines than that in late-bolting lines upon heat treatment (**Figure 2**). Knockdown of *LsFT* by RNAi resulted in delayed bolting in lettuce and failed to respond to heat treatment (**Figure 4**), suggesting the important role of *LsFT* during bolting regulation in lettuce (Fukuda et al., 2017). In lettuce, floral transition may require a *LsFT* threshold, and heat can accelerate such transition. The decreased *LsFT* expression observed in the late-bolting lines or RNAi lines may fail to reach the *LsFT* threshold and thus unable to perceive heat signal and resulted in late bolting. The opposite effect of heat treatment on flowering in lettuce and chrysanthemum may due to different response element in the *FT* promoter. In *Arabidopsis, CO* binds to the DNA via a special sequence element containing a consensus TGTG(N2-3)ATG motif that is present in tandem within the *FT* promoter (Tiwari et al., 2010). The MADS box genes, *FLC* and *SHORT VEGETATIVE PHASE (SVP)*, form a complex to repress *FT* expression through binding to the DNA regions within the proximal *FT* promoter and the first intron that contains CArG boxes (Adrian et al., 2010). Therefore, it would be intriguing to explore the heat-responsive element in the *LsFT* promoter in the future, which will shed light on the specific functions of *LsFT* during heat response in lettuce. Despite bolting is generally followed with flowering in lettuce, bolting and flowering are two separate processes and may be regulated by different mechanisms. More studies using floral markers such as *LsAP1* and *LsLFY* are needed to differentiate lettuce bolting and flowering in the future.

## Author Contributions

We thank members of the Zhang and Wang Labs for discussions and help with techniques. We also thank BioMed Proofreading LLC for proofreading this manuscript. ZC, XZ, and QW conceived and designed the experiments. ZC, YH, KN, YD, SY, WZ, CL, and XJ performed the experiments. ZC, YH, KN, and YD analyzed the data. DG and RL uploaded the database. ZC, XZ, and QW wrote the paper. All authors read and approved the final manuscript.

## Conflict of Interest Statement

The authors declare that the research was conducted in the absence of any commercial or financial relationships that could be construed as a potential conflict of interest.

## Footnotes

1 http://faostat3.fao.org/browse/Q/QC/E

2 http://www.arabidopsis.org/index.jsp

3 https://genomevolution.org/coge/
