# Supplementary material for: Inflorescence Development and the Role of LsFT in Regulating Bolting in Lettuce (Lactuca sativa L.)
Source: Front Plant Sci. 2018 Jan 18;8:2248. doi: 10.3389/fpls.2017.02248 (PMC5778503; doi:10.3389/fpls.2017.02248)
Supplement: Supplementary file 1 [file Table_1.PDF]

Supplemental Table S1. List of primers used in this study.

Gene	Forward primer (5'-3')	Reverse primer (5'-3')
**For gene amplification and vector construction**		
LsFT-cloning	ATGATGCCTAGGGAGAGGGACCC	TTATCTTCTTCGCCCACCAA
LsAP1-cloning	ATGGGAAGAGGAAGGGTGACGTT	TCACTTGTTCATGTGTTGAATC
LsAP3-cloning	ATGGGTAGGGGGAAGATAGAGA	TCAAGGAAGGCGATGATCATGAG
LsLFY-cloning	ATGGACCCTGAAACACTCTCGGC	CTAAAACTGGAGATGACCACCACC
LsFT-OE	GCTCTAGAATGATGCCTAGGGAGAGGGACCC	TCCCCCGGGTTATCTTCTTCGCCCACCAA
LsFT-RNAi-1s	TTGGCGCGCCCGTGTGATAGGAGATGTTCT	
LsFT-RNAi-1a	ATTTAAAT GATCGACCATCACTAA	
LsFT-RNAi-2s	GGACTAGTCGTGTGATAGGAGATGTTCT	
LsFT-RNAi-2a	CGGGATCCGATCGACCATCACTAA	
LsFT--PUC	GGACTAGTATGATGCCTAGGG	TCCCCCGGGTCTTCTTCGCCCACCAA
**For transgenic plants identification**		
CaMV35S	GACGCACAATCCCACTATCC	
OV-LsFT	TCCCCCGGGTTATCTTCTTCGCCCACCAA	
1008-S	CGTCTTCAAAGCAAGTGGATT	
1008-A	CGAAACCAATGCCTAAAGAGAG	
**For qRT-PCR**		
LsFT	GACAGTTTCACAAAGTCGATTAA	TGTGAAAAGCCCGGAGG
LsAP1	CTAGAGAAAGACATACCCTCCCAC	TCACTTGTTCATGTGTTGAATCA
LsAP3	TGCCGGAAGAGGGTGAAAA	TCAAGGAAGGCGATGATCATG
LsLFY	TCTGTCATGCTGAACGCAGC	CTAAAACTGGAGATGACCACCACC
18sRNA	GTGAGTGAAGAAGGGCAATG	AGTGAATTGGTTTCGAGAGC
AtACTIN2	CCTTCGTCTTGATCTTGCGG	AGCGATGGCTGGAACAGAAC
