# Supplementary material for: Inflorescence Development and the Role of LsFT in Regulating Bolting in Lettuce (Lactuca sativa L.)
Source: Front Plant Sci. 2018 Jan 18;8:2248. doi: 10.3389/fpls.2017.02248 (PMC5778503; doi:10.3389/fpls.2017.02248)
Supplement: Supplementary file 2 [file Table_2.PDF]

Table S2. Gene expression levels (Transcripts per million) of flowering genes in the SAM of lettuce line S39 at different stages isolating by laser capture microdissection and detected by RNA-Seq.

ID (Model name)	Stage 1	Stage 2	Stage 3	Stage 4
Last_1_v5_gn_7_6780 (LsSOC1)	25±2	342±42	1142±258	1406±371
Lsat_1_v5_gn4_ 84380 (LsLFY)	22±1	28±10.5	147±21.5	19678±287

Note: values are means of two biological replicates, ±sd.
