# Supplementary material for: Inflorescence Development and the Role of LsFT in Regulating Bolting in Lettuce (Lactuca sativa L.)
Source: Front Plant Sci. 2018 Jan 18;8:2248. doi: 10.3389/fpls.2017.02248 (PMC5778503; doi:10.3389/fpls.2017.02248)
Supplement: Supplementary file 3 [file Data_Sheet_1.PDF]

Inflorescence development and *LsFT* regulation of heat-induced flowering in lettuce (Lactuca *sativa*L.)

Zijing Chen[1,*], Yingyan Han[2,*], Kang Ning[1], Yunyu Ding[1], Wensheng Zhao[1], Shuangshuang Yan[1], Chen Luo[1], Xiaotang Jiang[1], QianWang[1, #], Xiaolan Zhang[1, #]

Figure S1

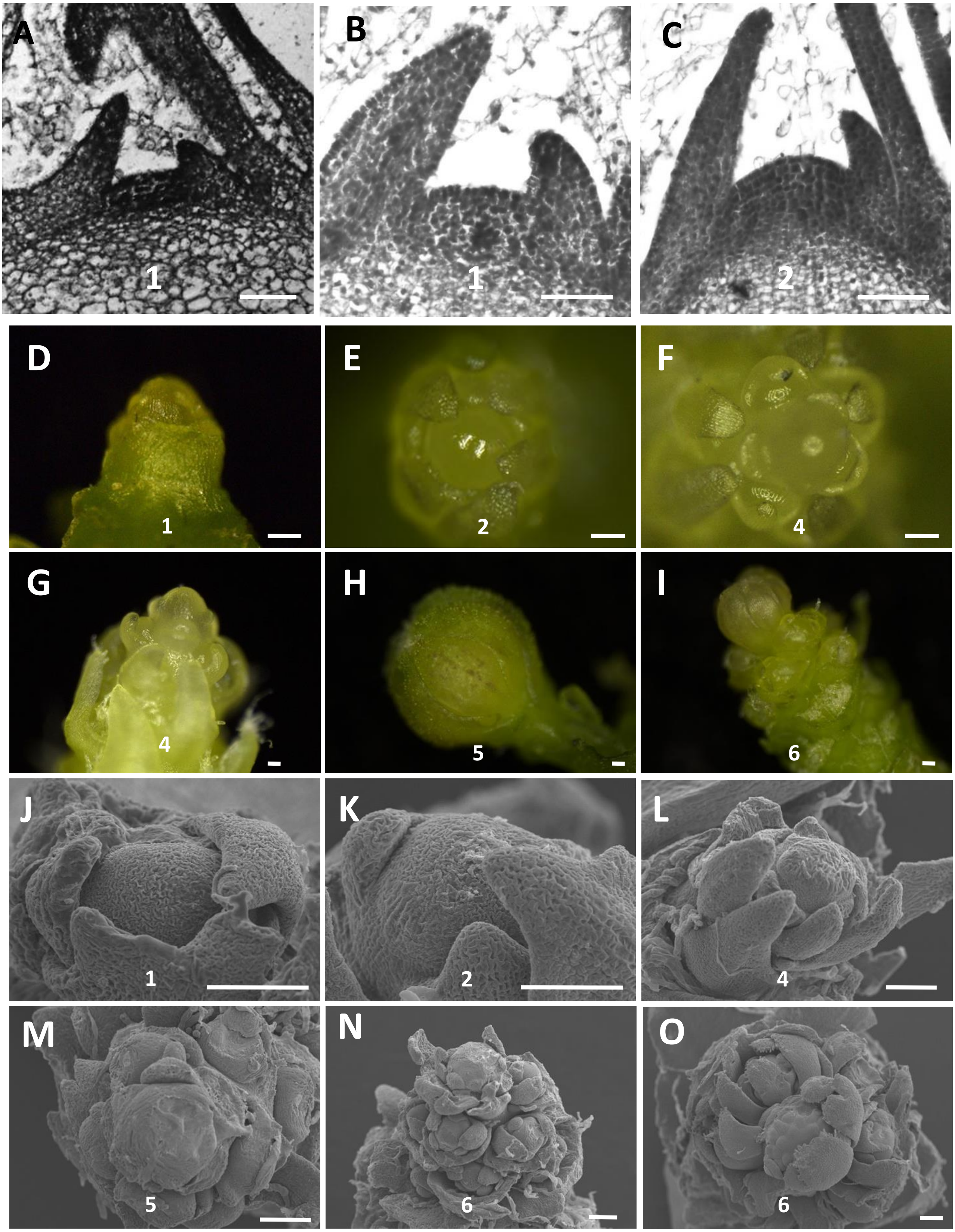

Supplemental Figure S1. Meristem images during floral transition in lettuce.

(A) Vegetative shoot apical meristem (SAM) of bolting resistant line S24 at 20 DAP [days after planting]). (B) Vegetative shoot apical meristem SAM of line S24 at 28 DAP. (C) Dome-shaped SAM of line S24 at 45 DAP. (D–I) Meristem images of line S39 taken by optical microscope. (D) Vegetative SAM at stage 1. (E) Dome-shaped SAM at stage 2. (F-G) IM at stages 4. (H–I) IM at stages 5 (H) and stage 6 (I). (J–O) Meristem images of S39 taken by scanning electron micrographs. (J) Vegetative SAM at stage 1. (K) Dome-shaped SAM at stage 2. (L) Stage 4 meristem showing involucre primordia. (M–O) Capitulum primordia at stages 5 (M) and 6 (N-O). Scale bars represent and 50 µm in (A–C),200 µm in (D–I), 100 µm in (J–O)

Figure S2

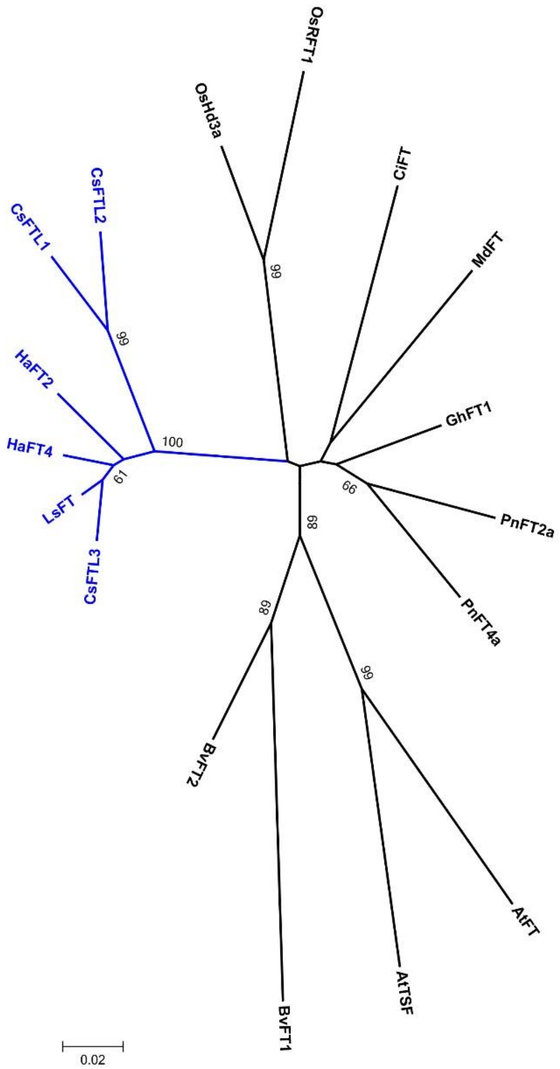

Supplemental Figure S2. Phylogenetic analysis of LsFT like proteins across species. The phylogenetic tree was constructed using the neighbor-joining method in MEGA 6 software. The scale bar represents 0.02 substitutions per site. The numbers next to nodes are 1,000 bootstraps. The blue represents FTs in the Asteraceae family. At: Arabidopsis thaliana; Cs: Chrysanthemum seticuspe; Ls : Lactuca sativa; Ha: Helianthus annuus. Os: Oryza sativa; Gh: Gossypium hirsutum; Md: Malus domestica; Ci: Citrus sinensis; Bv:Beta vulgaris; Pn: Populus nigra.

Figure S3

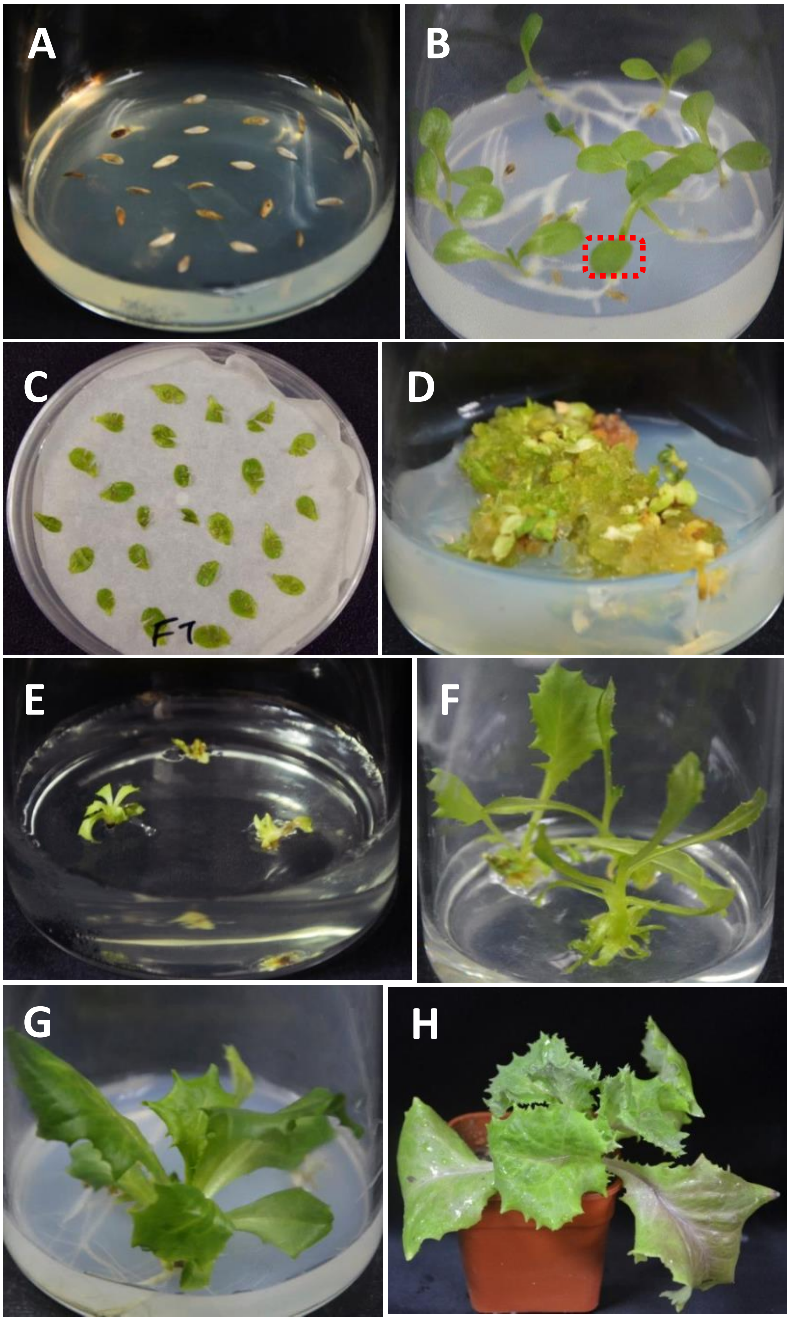

Supplemental Figure S3. Agrobacterium-mediated transformation in lettuce.

(A) Sterilized lettuce seeds sown on MS medium. (B) Cotyledons from 6-day-old seedlings that were ready for cutting. (C) Cut cotyledons were co-cultivated with agrobacterium on co-cultivating medium. (D) Green shoots formed from pale green calli in differentiation medium after three weeks. (E) Green shoots were transferred to the shoot-inducing medium. (F) Shoots developed two weeks after being transferred to shoot-inducing medium. (G) A regenerated shoot was transferred to the root-inducing medium, and roots formed two weeks after transfer. (H) After acclimation for one week, a regenerated plant was transferred to soil.
